# Morphing polymer voxels for dynamic information switching

**DOI:** 10.1126/sciadv.aeg9993

**Published:** 2026-09-25

**Authors:** Lijun Cai, Zhangming Shen, Wang Zhang, Hongtao Wang, Joel K. W. Yang, Hong Liu, Mingchao Zhang

**Affiliations:** ^1^ Department of Materials Science and Engineering, National University of Singapore, Singapore 117575, Singapore.; ^2^ Engineering Product Development, Singapore University of Technology and Design, Singapore 487372, Singapore.; ^3^ Singapore-HUJ Alliance for Research and Enterprise (SHARE), The Smart Grippers for Soft Robotics (SGSR) Programme, Campus for Research Excellence and Technological Enterprise (CREATE), Singapore 138602, Singapore.; ^4^ Institute of Materials Research and Engineering, Agency for Science, Technology, and Research (A*STAR), Singapore 138634, Singapore.; ^5^ School of Materials Science and Engineering, Nanyang Technological University, Singapore 639798, Singapore.

## Abstract

Conventional print is noninteractive, whereas modern electronic displays, even with improved energy efficiency, require continuous power input and generate electronic waste. Here, we report morphing polymeric voxels based on liquid crystal that enable seamless and reversible information display without onboard electrical power or integrated circuitry. Each voxel exhibits programmable dual birefringence arising from geometry-defined structural anisotropy and mesogen-defined material anisotropy. By mapping laser-writing angle and power, two distinct images are encoded within a single array. Mechanical deformation induces voxel buckling and hybrid director fields, reversibly switching brightness dominance between the two birefringence channels. This deformation-driven mechanism enables reversible transitions between preencoded images. Comprising only polymers and relying on passive optical modulation, the platform establishes a sustainable route toward circuit-free dynamic displays.

## INTRODUCTION

Popular fiction often depicts newspapers with animated headlines that update themselves in real time, with the magic newspaper “*Daily Prophet*” in *Harry Potter* as a familiar example (*1*). In reality, conventional print, made of cellulose and carbon inks, is intrinsically static: It reflects ambient light with high readability (passive reflective display) but cannot alter content once printed (*2*–*4*). Over recent decades, dynamic and up-to-date information has migrated to networked electronic displays (*5*–*7*). They provide immediacy, yet they rely on continuous energy, depend on critical materials, and inevitably contribute to ever-growing e-waste (*8*–*12*). This contrast motivates a simple question: Can we create a “*Daily Prophet*”–like print, with seamless and reversible switching between stories, without electronics, batteries, or circuits?

Recent advances in stimuli-responsive soft materials offer a path forward. By coupling optical state to ambient triggers such as light (*13*, *14*), heat (*15*–*17*), humidity (*18*, *19*), or chemical cues (*20*–*22*), these smart materials can reconfigure appearance with minimal external energy input. Despite this promise, many demonstrations remain binary (on-off modulation), with content that simply appears or disappears (*23*–*25*). As a result, they cannot achieve true multistate switching comparable to modern electronic displays. A central challenge is to decouple encoding from selection on demand. Smart materials that are easy to encode often lack independent and multistate selection, whereas systems that switch readily are difficult to encode without cross-talk (*26*–*29*). In soft optical materials, contrast typically arises from structural effects governed by geometry or from material anisotropy governed by molecular orientation (*30*–*35*). If both pathways could be programmed within the same voxel, and if selection could be achieved mechanically and seamlessly, richer state spaces could be accessed without electrical addressing.

Here, we introduce morphing polymer voxels that allow seamless and reversible information switching without onboard electrical power or integrated circuitry. Each voxel is a uniaxially aligned liquid crystal network (LCN) with programmable dual birefringence. The platform uses orientated liquid crystal mesogens that are readily cross-linked by two-photon polymerization (2PP) as the voxel units. These voxels exhibit structural birefringence, arising from the printed geometry, and material birefringence, arising from the mesogen director. Structural birefringence produces angle-dependent brightness at orthogonal azimuth in a crossed polarizers configuration (CPC), which enables pattern programming by adjusting the writing angle during 2PP. Material birefringence produces height-dependent colors at nonorthogonal azimuth under CPC, which provides a second programming route by tuning the exposure dose. By integrating these two logic states (writing angle for structure and laser dose for material anisotropy), two distinct images can be encoded in a single printed array, with switching achieved by simple sample rotation under CPC. To eliminate manual rotation and achieve in situ switching, we apply a global compression to deform the printed array voxels, which enables spontaneous mesogen reorientation. This reversible deformation swaps the dominant optical contribution between structural and material birefringence, producing seamless transitions between the angle-encoded and dose-encoded images. Overall, the fully polymeric display platform supports complex information storage and dynamic image display, advancing next-generation, energy-efficient optical devices and moving a step toward “magic” newspaper.

## RESULTS

### Concept of the all-polymer display platform

**Fig. 1.**
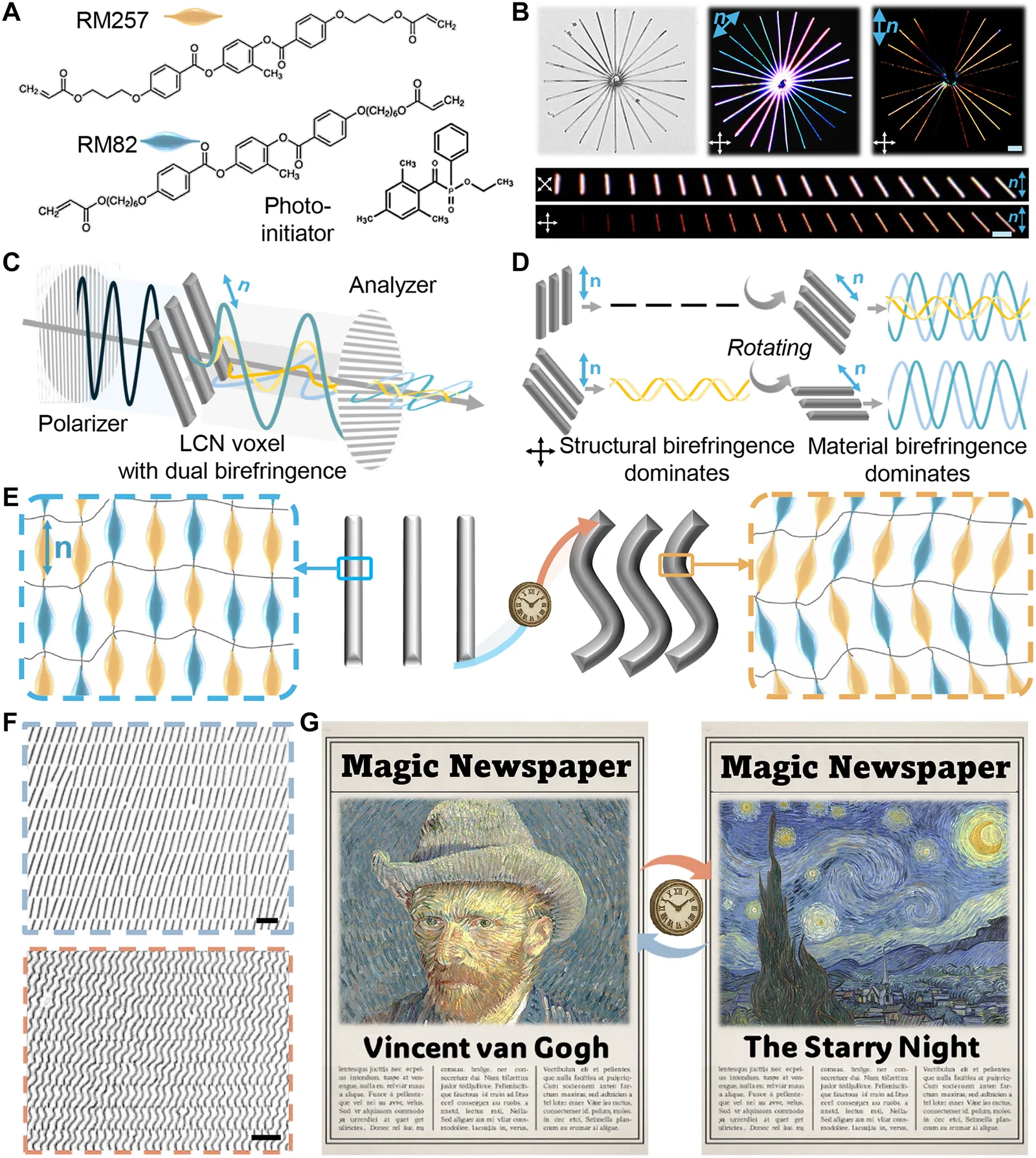
Concept of image switching based on morphing polymer voxels. (**A**) Main precursors of the nematic mesogen resist. (**B**) Optical images showing LCN filaments written at different in-plane orientation. The mesogen director is marked by vector **n** in blue. The azimuth of CPC was marked by the white crossed arrows. Scale bars, 10 μm. (**C**) Schematic of polarized light passing through an LCN voxel. Under CPC, incident linearly polarized light is resolved into ordinary and extraordinary components within the anisotropic LCN voxel. The brightness is determined by the effective phase retardation arising from two independent sources: material birefringence defined by the liquid crystal director (**n**) and structural birefringence defined by the hatching angle. (**D**) Schematic of rotating the LCN voxel toggles the brightness-dominant channel. Channel dominance depends on orientation relative to the CPC. When both the LC director** n** and the hatching angle are orthogonal to the CPC, neither material nor structural birefringence generates effective retardation under crossed polarizers, and the voxel appears dark. When both are oblique, both channels contribute finite retardation and the voxel appears bright. When **n** is orthogonal but the hatching angle is oblique, material birefringence is suppressed and structural birefringence dominates. Conversely, when the hatching angle is orthogonal but **n** is oblique, structural birefringence is minimized and material birefringence governs the brightness. Structural birefringence is weaker in magnitude than material birefringence. (**E**) Scheme of LC orientation and voxel geometry before and after deformation. (**F**) Optical images of voxel arrays before and after deformation. Scale bars, 10 μm. (**G**) Schematic illustration of seamless and reversible image switching; the newspaper templates serve as the schematics.

Each voxel is a uniaxially aligned (nematic) LCN that exhibits two optical channels: material birefringence set by the mesogen director and structural birefringence set by the printed geometry. Nematic LCN structures fabricated through 2PP process serve as the voxel units (Fig. 1A). A voxel is bright when its mesogen director is neither parallel nor perpendicular to the CPC since material birefringence introduces phase retardation between ordinary and extraordinary rays (Fig. 1B). Voxel height sets the optical path difference, so height variations yield distinct interference colors. We found that the 2PP laser dose can also control voxel height and therefore tune visible spectra (colors). In contrast, when the mesogen director is parallel or perpendicular to the CPC, the phase retardation vanishes and brightness drops. The printed filaments with aspect ratio >1 also display a fourfold symmetric angular response in brightness, with maxima at 45° + *n* × 90° and minima at 0° + *n* × 90° (*n* is an integer). The geometric asymmetry of the printed structures generates spatially varying refractive indices and thus structural birefringence, which dominates near the material-birefringence minima, providing a second, non–cross-talk route to program angle-dependent brightness. Voxel optics arise from both geometry and molecular alignment and rotation shifts which contribution dominates the brightness (Fig. 1, C and D).

By selecting the laser dose and laser writing angle to program the material and structural contribution to brightness, two distinct images can be encoded into a single printed array. Simple sample rotation under CPC selects between the two images, but rotation requires manual intervention and does not provide autonomous playback. To achieve in situ channel swapping, we sought to find strategy to twist the voxel units during operation as we deem the local director departs from the orthogonal orientations when voxels twist, and the brightness contribution governed by structural birefringence can be overtaken by that governed by material birefringence. To twist the voxels, we apply a uniform global compression by embedding them into a phase change matrix on actuation (Fig. 1E). Compression deforms the structure, reorienting the mesogen director and shifting brightness dominance from structural to material birefringence, which switches the displayed image without manual rotation (Fig. 1, F and G). Notably, cooling releases the compression, the array unbuckles, and the director returns to its initial alignment, restoring structural birefringence as the dominant channel. The process is reversible, enabling seamless complex image switching.

### 2PP fabrication of LCN voxels with dual birefringence

**Fig. 2.**
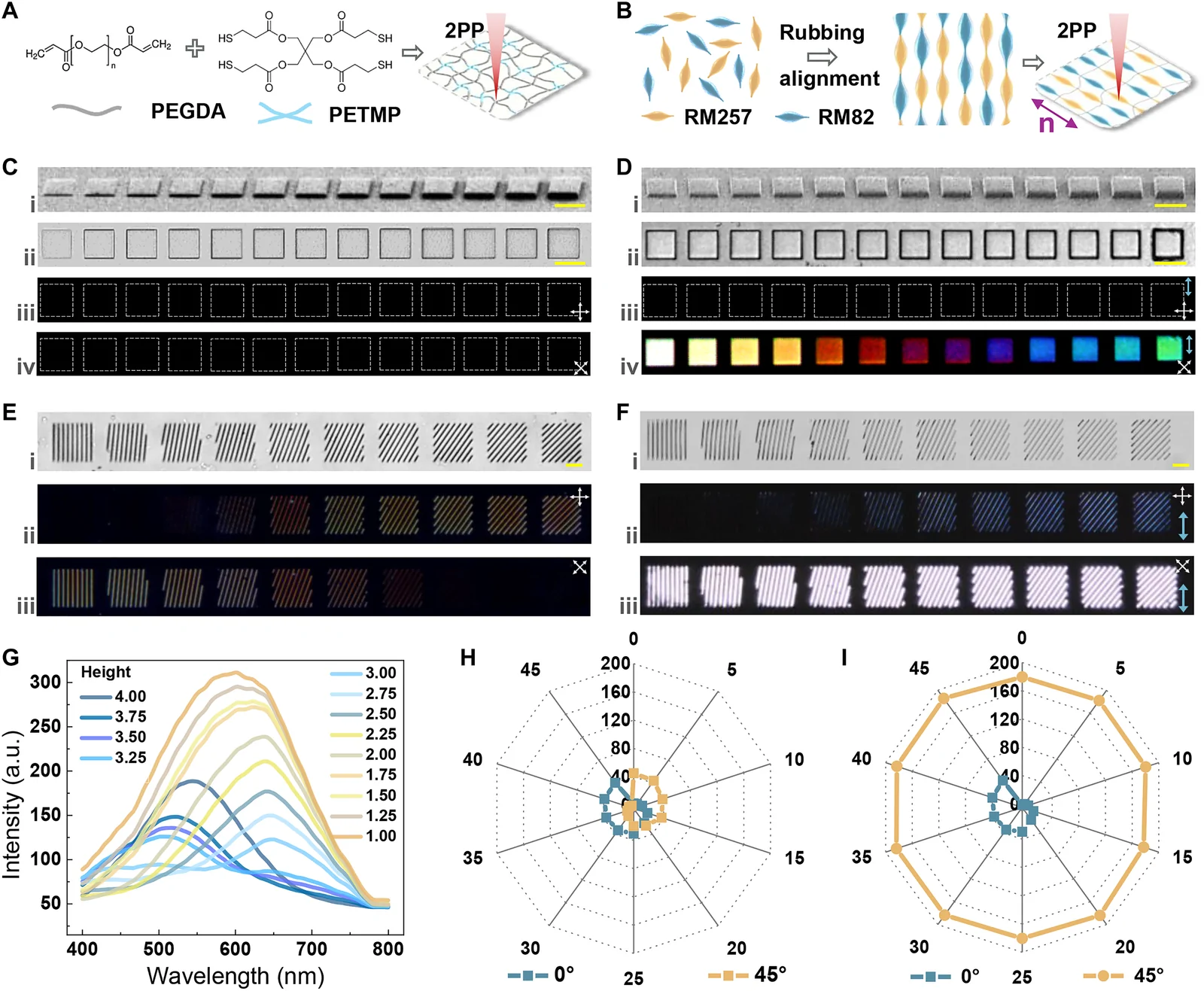
LCN voxels with dual birefringence. (**A** and **B**) Illustration of PEGDA voxels (A) and LCN voxels (B). (**C** and **D**) SEM image (i), bright-field image (ii), polarized optical image at 0° azimuth CPC (iii) and 45° azimuth CPC (iv) of PEGDA voxels (C) and LCN voxels (D) with height varied from 1 to 4 μm. Scale bars, 20 μm. (**E** and **F**) Bright-field image (i), polarized optical image under 0° CPC (ii) and 45° CPC (iii) of PEGDA fins (E) and LCN fins (F). Scale bars, 20 μm. (**G**) Spectrum of LCN cubes with height varied from 1 to 4 μm. (**H** and **I**) Brightness analysis of PEGDA fins (H) and LCN fins (I) at 0° and 45° azimuth CPC. a.u., arbitrary unit.

LCN voxels were fabricated by 2PP of nematic LC mesogens, and they exhibited both material and structural birefringence. This contrasts with isotropic polymer, which can show structural birefringence from geometry but lack material birefringence. To benchmark the response, we printed randomly arranged polymer network [polyethylene glycol diacrylate (PEGDA) network, Fig. 2A] and uniaxially aligned LCN voxels {formulated form 1,4-Bis-[4-(3-acryloyloxypropyloxy)benzoyloxy]-2-methylbenzene (RM257) and *1,4-Bis-[4-(6-acryloyloxyhexyloxy)benzoyloxy]-2-methylbenzene* (RM82); Fig. 2B} using the same 2PP process. Cubes of varying height were written from each resist and compared optically. Under CPC, PEGDA cubes remained dark at all azimuths, consistent with isotropic polymer chains and thus isotropic optics (Fig. 2C). In contrast, LCN cubes appeared dark at orthogonal CPC and showed vivid colors at other azimuths (Fig. 2D and fig. S1), reflecting optical anisotropy from uniaxial alignment (nematic) of mesogens. When linearly polarized light traverses a birefringent nematic material, a phase retardation (δ) forms between the ordinary and extraordinary rays, and thus resulting in variation in the optical path of the transmitted light (*36*–*39*). The δ can be expressed as

$$\delta =2\pi \left(n_{e}-n_{o}\right)\cdot d/\lambda$$ (1)

where *n*_e_ and *n*_o_ are the extraordinary and ordinary refractive indices, *d* is the voxel thickness, and λ is the wavelength. Increasing *d* changes the optical path difference, which selects wavelengths by interference and produces distinct interference colors (Fig. 2, D and G).

When the printed structures were designed as filaments (aspect ratios >1) and written at different in-plane laser angle, both isotropic PEGDA and nematic LCN filamentary voxels (fins) showed angle-dependent brightness even at orthogonal CPC (Fig. 2, E and F). As shown in Fig. 2 (E-ii and F-ii) and fig. S2, the brightness varied with writing angle: Fins written at 0° or 90° appear dark, whereas those written 45° were maximally bright. This behavior arises from their geometry anisotropy, which induces spatially varying refractive indices and optical path differences and, therefore structural birefringence (*33*, *40*, *41*). To be specific, when polarized light traverses these anisotropic fins, it splits into two orthogonally polarized components that experience distinct effective refractive indices. The resulting phase retardation between the two components yields residual transmission through the analyzer, resulting in angle-dependent optical performance. Placing the sample at 45° azimuth reversed the trend in PEGDA fins (Fig. 2E, iii, and fig. S2C). A pattern written at 45° becomes parallel to one polarizer at this viewing geometry and darkens, while a pattern written at 0° becomes oblique and brightens. This swap confirms that the brightness originates from the relative orientation between the microfilaments and the polarization axes, consistent with a purely structural origin (Fig. 2H). In contrast, LCN fins became uniformly bright at 45° CPC (Fig. 2F, iii, and fig. S2B). Once the viewing angle departs from orthogonal CPC, material birefringence of the LCN dominated and overwhelmed the weaker structural contribution, so the brightness differences between writing angles largely vanished (Fig. 2I). These results show that the periodic LCN fins have dual birefringence, with structural and material channels that can be addressed by viewing geometry.

### Independent information encoding in two channels

**Fig. 3.**
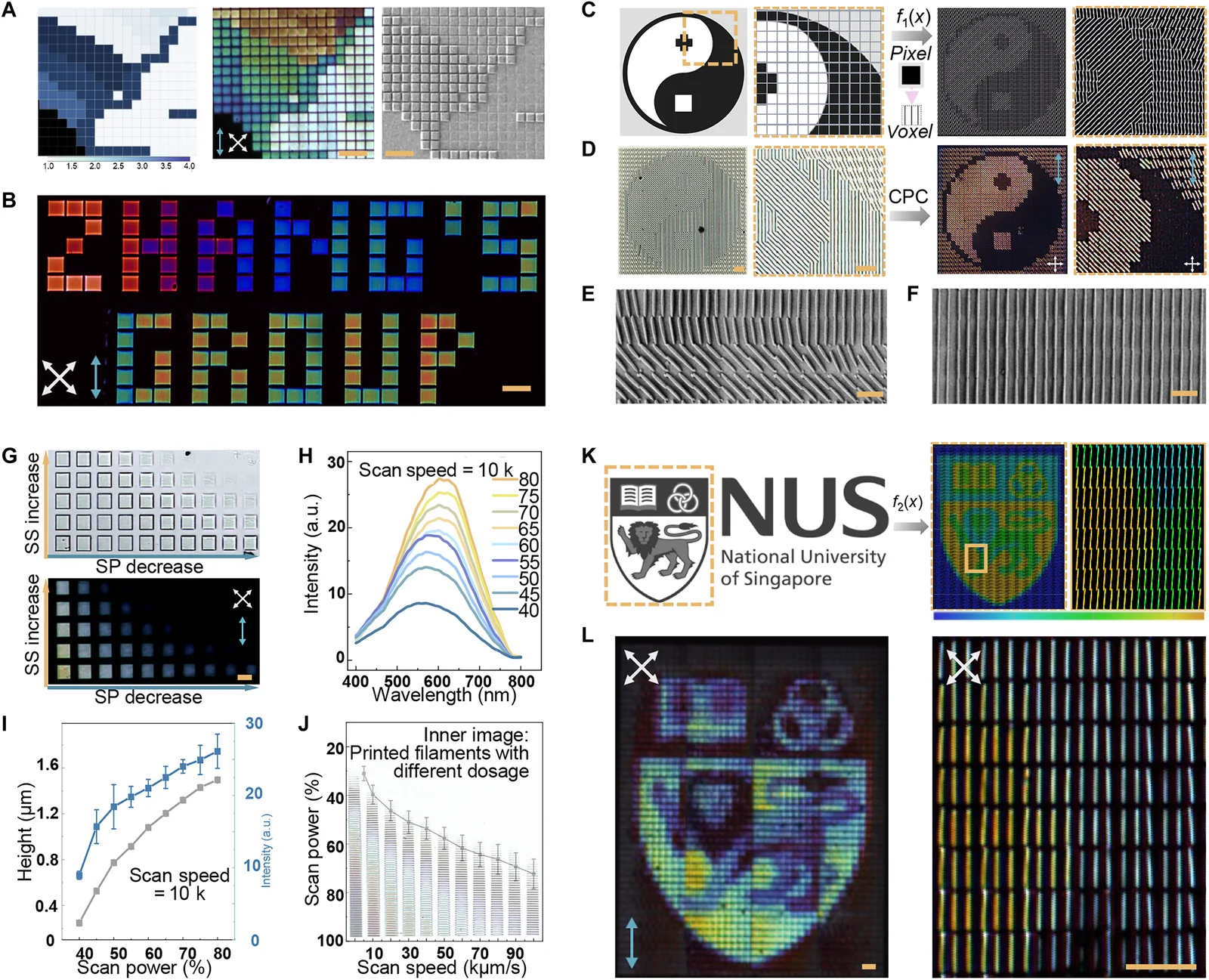
Single-channel programmability of LCN voxels. (**A**) Layout (left), optical image (middle), and SEM image (right) of the printed array with cubes in different heights. Scale bars, 60 μm. (**B**) Reflective optical microscope image of sample viewed under polarized illumination with an orthogonal analyzer showing colorful ZHANG’S GROUP pattern. Scale bar, 60 μm. (**C**) Programming logic diagram of writing angle–modulable Tai Chi diagram including the pixelation process (left) and program overview from DeScribe (right). (**D**) Bright-field image (left) and polarized image (right) of printed Tai Chi diagram. Scale bars, 20 μm. (**E**) SEM images of printed fin arrays. Scale bar, 10 μm. (**F**) SEM images of the printed NUS emblem fin arrays. Scale bar, 10 μm. (**G**) Bright-field image (top) and polarized image (bottom) showing LCN voxels printed with different laser dosage. Scale bar, 20 μm. (**H**) Spectrum of the LCN voxels printed with SS of 10 kμm/s and different SP. (**I**) Height and intensity of LCN voxels printed with SS of 10 kμm/s and different SP. (**J**) Printability of LC resist with NVP. (**K**) Programming logic diagram of dosage-modulable NUS emblem including the pixelation process (left) and program overview from DeScribe (right). (**L**) Polarized image of THE printed NUS emblem. Scale bars, 20 μm. a.u., arbitrary unit.

Because the optical performance of LCN voxels can be modulated by their geometry, we tried to program them. At nonorthogonal CPC, the color of an LCN cube voxel depends on height. We thus printed an array of voxels in different heights (Fig. 3A), which produced a multihued pattern. Scanning electron microscopy (SEM) image showed height variations across the array, leading to the corresponding optical path differences. By programming voxels in different heights, a colorful “ZHANG’S GROUP” pattern was obtained (Fig. 3B). In parallel, the LCN fins provide an angle-addressable structural channel: Assigning different writing angles to different voxels renders a brightness map at orthogonal CPC. A grayscale Tai Chi diagram was mapped by a function *f*_1_(*x*) that linearly converts normalized gray level to writing angle in the range 0° to 45° (Fig. 3C). As each voxel showed angle-dependent brightness, a Tai Chi diagram could be constructed at orthogonal CPC (Fig. 3D). SEM images further confirmed that fins could be written in different angles for constructing patterns (Fig. 3E).

Laser dose has influence on voxel height and thus on color and brightness (spectra). Decreasing scan power (SP) at a fixed scan speed (SS) produced a gradual blue shift and reduced brightness with decreasing dose (Fig. 3G), while increasing SS at a fixed SP induced similar spectral shifts (Fig. 3H), confirming that exposure dose governs the spectra (fig. S3). Atomic force microscopy showed a monotonic decrease in voxel height with decreasing dose (Fig. 3I and fig. S4), attributable to reduced photon flux and a smaller fraction of the focal volume exceeding the polymerization threshold (*42*, *43*). This trend was geometry independent and also appeared in fins (fig. S5). We mapped the printability window systematically to understand the laser dosage boundary. At *SS* = 10 kμm s^−1^, stable LC microstructures formed for *SP* = 25 to 50 mW (fig. S6). At *SS* = 100 kμm s^−1^, powers even above 45 mW were still insufficient for continuous features. To extend the window, we added *N*-vinylpyrrolidone (NVP) to the LC resist, which could lower viscosity, enhance diffusion during polymerization, and suppress local overheating at high powers (*44*, *45*). Thus, NVP broadened the usable dose range and improved structural fidelity, enabling high-quality reconstructions (Fig. 3J). Within this, using a mapping *f*_2_(*x*) from gray level to SP within 25 to 50 mW, we fabricated a discretized National University of Singapore (NUS) emblem with high contrast and edge definition (Fig. 3, F, K, and L).

### Dual-channel programming

**Fig. 4.**
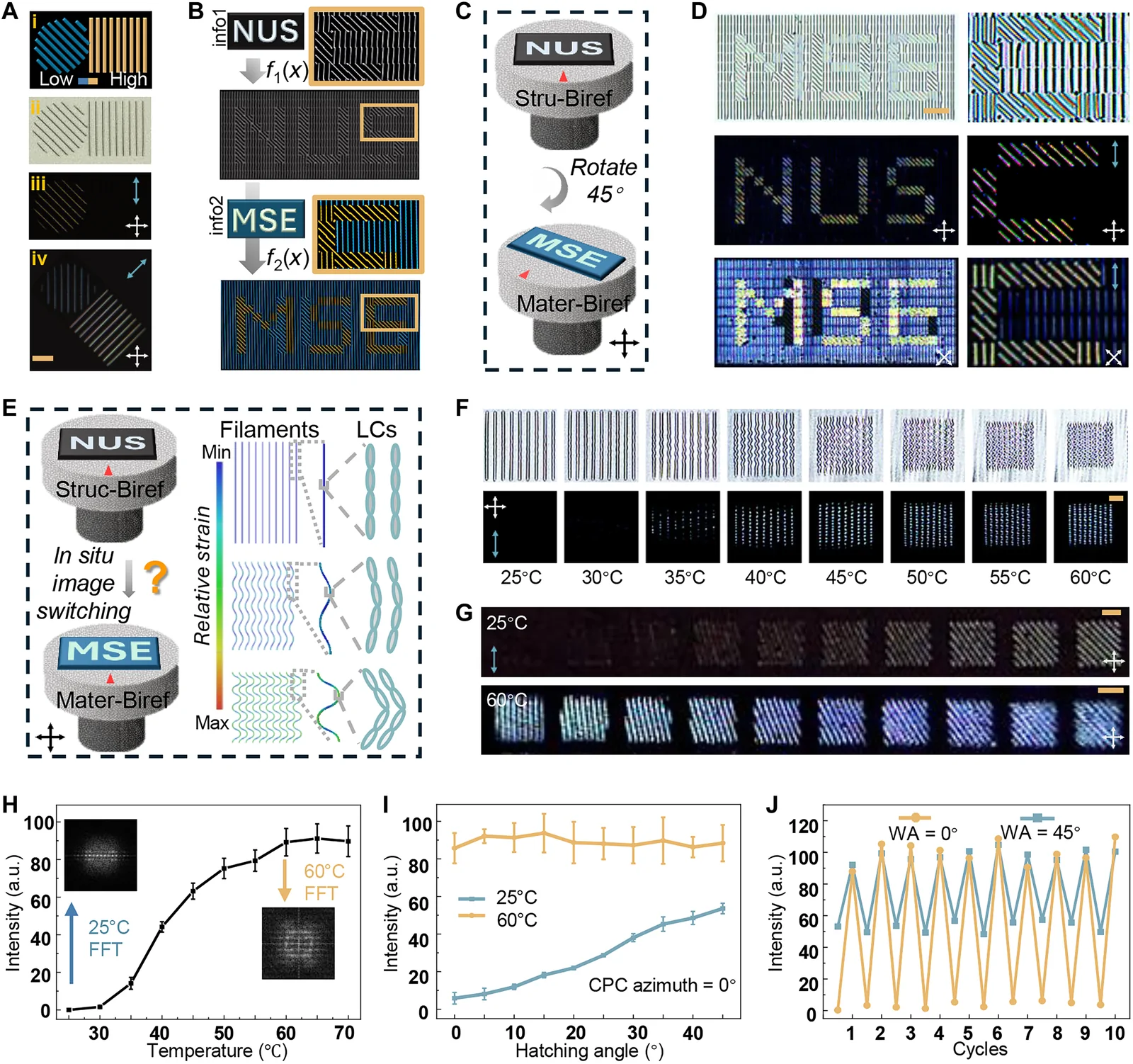
Dual channel programmability and in situ switching of LCN voxels. (**A**) Conceptive proof of dual channel programmability including program overview from DeScribe (i), bright-field image (ii), polarized images of printed motifs before (iii), and after (iv) rotation. Scale bar, 20 μm. (**B**) Programming logic diagram of simultaneously encoding two images within one fin array. (**C**) Schematic illustration of rotation-induced image switching. (**D**) Bright-field image, polarized image of voxel array with dual channel encoded before and after sample rotation. Scale bar, 10 μm. (**E**) Schematic illustration of in situ image switching (left) and FEA results of compressive force on filaments and corresponding LC orientation (right). (**F**) Bright-field images (to) and polarized images (bottom) of LCN fin embedded in hydrogel upon heating process. Scale bar, 10 μm. (**G**) Polarized images of fin arrays with different writing angle embedded in hydrogel at 25°C (top) and at 60°C (bottom). Scale bars, 20 μm. (**H**) Brightness analysis of LCN fin embedded in hydrogel during heating process. The inner images are FFT magnitude spectrum of LCN cube at 25° and at 60°C. (**I**) Brightness analysis of hydrogel-embedded fin arrays with different writing angle at 25° and at 60°C. (**J**) Brightness analysis of LCN fin with writing angle (WA) of 0° and 45° during heating-cooling circles. a.u., arbitrary unit.

Because structural birefringence dominates at orthogonal CPC and material birefringence dominates at other azimuths, the two optical channels are naturally decoupled and free of cross-talk. This enables concurrent encoding of information in a single LCN fin by programming writing angle for the structural channel and laser dose for the material channel. As a proof of concept, we printed fins with two geometries: a round motif written at 45° using a lower laser dose and a square motif written at 0° using a higher dose (Fig. 4A, i). At orthogonal CPC, the round motif appeared bright while the square motif was dark, consistent with structural birefringence selecting by relative geometry (Fig. 4A, iii). Rotating the sample to a nonorthogonal CPC activated material birefringence, and both motifs brightened with distinct colors, with round voxel appearing blue and square voxel presenting yellow (Fig. 4A, iv). These observations confirm concurrent control of brightness and color for dual channels of complex information through writing angle and laser dose within a single voxel.

We then extended this strategy to fin arrays with two simultaneously encoded images. For the “NUS” channel, a function *f*_1_(*x*) assigned writing angles from normalized gray levels. For the “MSE” channel, another function *f*_2_(*x*) assigned SP within the printable window to the corresponding voxels (Fig. 4B). The printed array displayed NUS at orthogonal CPC and, upon manual rotation to a nonorthogonal CPC, transitioned seamlessly to MSE (Fig. 4, C and D). Returning to orthogonal CPC restored NUS, demonstrating fully reversible and repeatable switching between two independently encoded images. The same dual-channel method produced other switchable motifs, such as the Tai Chi diagram (fig. S7).

### Design and mechanism of in situ switching

Sample rotation can select between the two channels but still requires external manipulation. We therefore searched for an approach that enables seamless and in situ switching within the printed voxel arrays themselves. The design principle is to introduce a stimulus that reorients the mesogen director away from its initial alignment parallel to orthogonal CPC. Reorientation activates material birefringence and shifts brightness dominance from the structural channel to the material channel, allowing a switch from an angle-encoded image to a dose-encoded image without mechanical rotation. However, reorientating the mesogen without causing rupture to the voxels is technically challenging at such small scales, and this led us to reconsider the problem from a mechanics standpoint and to ask whether controlled, reversible deformation could provide the needed local director reorientation without compromising their structural integrity.

Guided by this motivation, we built finite-element models of our high-aspect-ratio fins and found that uniform in-plane compression concentrates axial strain energy until a critical force $F_{\text{cr}}$ is reached (Fig. 4E). Beyond this threshold, the straight configuration becomes unstable, and the filaments buckle laterally, twisting the fins and deflecting the director away from orthogonal CPC. To realize this behavior uniformly across large areas, we finally came up a strategy by embedding the arrays in an optically transparent thermoresponsive hydrogel that shrinks controllably on heating (fig. S8). Hydrogel matrix shrinkage applies uniform compression, drives buckling once $F>F_{\text{cr}}$ (*46*). This process changed the fin geometry, eliminating the original optical anisotropy led by structural birefringence. Simultaneously, the buckling perturbs the liquid crystal director field, causing it to deviate from the orthogonal azimuth to CPC and thereby activating the material birefringence contribution. This shifts brightness dominance from the structural to the material channel. Under heating, fins written at 0° progressively deform (Fig. 4F), and fast Fourier transform (FFT) analysis of their profiles showed spot broadening and splitting, consistent with reduced periodicity and the emergence of multidirectional features during buckling (fig. S9). The corresponding FFT angular spectra broadened with temperature, confirming director reorientation. Concomitantly, their optical state at orthogonal CPC changed from dark to bright, indicating a shift of dominance from structural to material birefringence (Fig. 4F). Quantitative analysis showed that brightness increases from 25° to 60°C and then saturates, so 60°C was used as the operating temperature for reliable switching (Fig. 4H). The compression-induced protocol yielded the same brightness and color switching for the round and square motifs as in the rotation-induced case (fig. S10 and Fig. 4A). Fins printed at different doses maintain their distinct color identities after switching (fig. S6).

Simultaneous deformation of the fins written at different angles further confirms in situ channel swapping under hydrogel-induced compression. At 25°C and orthogonal CPC, the arrays displayed the expected angle-dependent brightness. Heating to 60°C yielded a uniformly bright state across all writing angles (Fig. 4I and movie S1). This trend mirrors the behavior observed when manually rotating the sample from 0° to 45° CPC (fig. S11). Cooling to 25°C restored the original angle-dependent contrast, and reheating returned the arrays to the bright state. Over at least 10 thermal cycles, voxels written at 0° and 45° showed stable, repeatable brightness modulation with minimal drift (Fig. 4J). The images of fin arrays after the 10th heating-cooling cycles further demonstrated the reversibility (fig. S12). We attribute this durability to the elastic LCN network, which provides decent reversible deformation and shape recovery. Together, these results establish hydrogel-induced buckling as a robust route to reversible switching between the two independently encoded channels.

### Seamless and in situ image switching

**Fig. 5.**
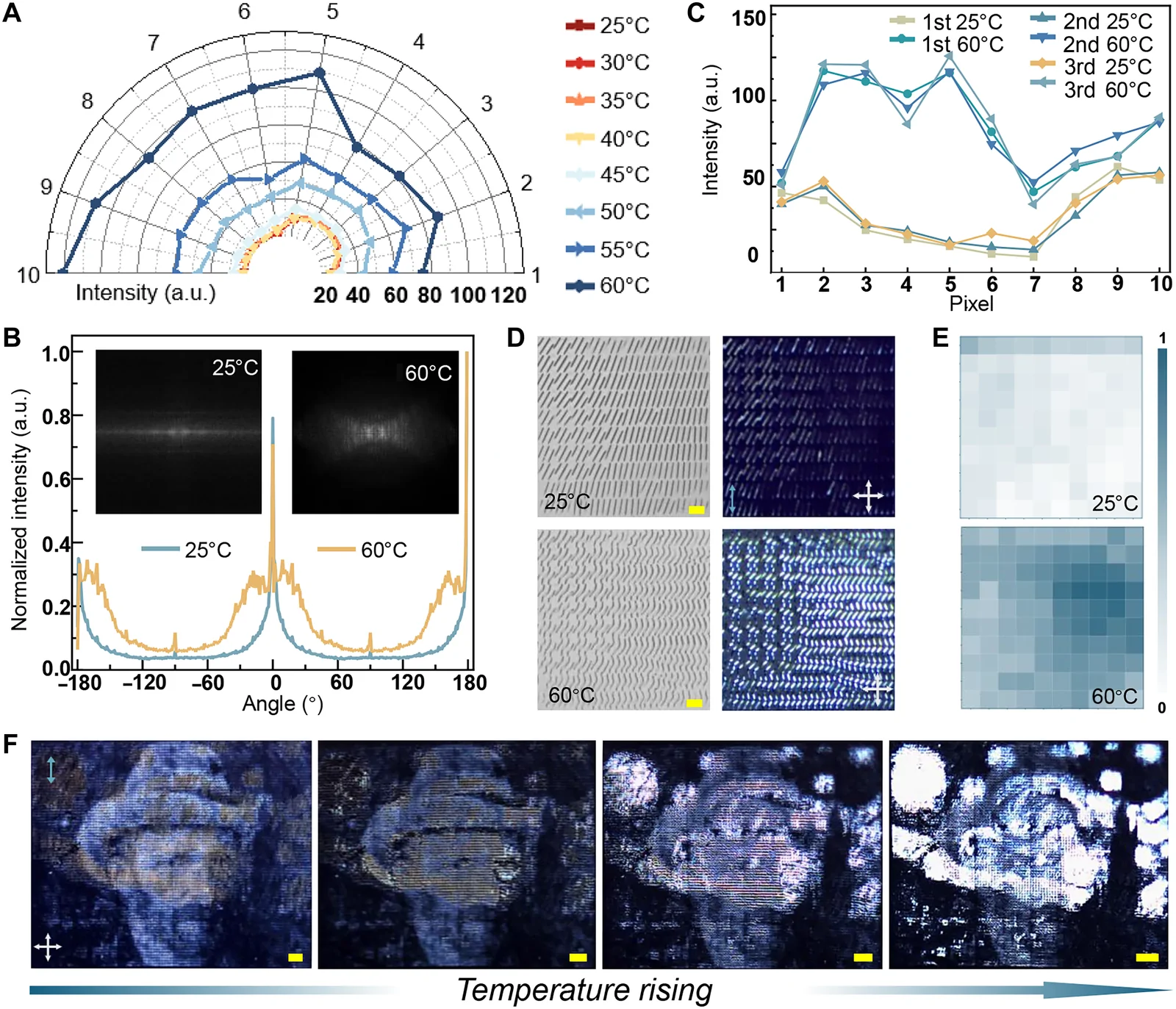
Programable, seamless, and reversible image switching. (**A**) Brightness analysis of 10 voxels in the printed array upon heating process. (**B**) Angular FFT of selected printed arrays at 25° and 60°C. Inner images are their corresponding FFT magnitude spectrum. (**C**) Brightness analysis of 10 voxels in the printed array upon heating-cooling process for three cycles. (**D**) Enlarged bright-field images (left) and polarized images (right) of the printed array encoded with two images at 25° and 60°C. Scale bars, 10 μm. (**E**) Brightness heatmap of the two enlarged polarized optical images at 25° and 60°C. (**F**) Polarized images showing the van Gogh’s self-portrait gradually switched to the *Starry Night* with temperature rising. Scale bars, 50 μm. a.u., arbitrary unit.

Thermally driven deformation, combined with dual-channel programmability, enables programmable and seamless information switching. We used a 10 μm–by–10 μm square LCN fins as the voxel, whose voxel density reached 10^6^ voxels cm^−2^. The intravoxel hatching distance was then systematically optimized. When the spacing was <3 μm, excessive bending and filament self-contact occurred during actuation, producing uncontrolled deformation, nonuniform brightness, and reduced voxel fidelity (fig. S13). Balancing mechanical stability and optical uniformity, we selected a 3.3-μm hatching distance for subsequent images. Using the optimized voxels, we randomly printed an array with different powers and angles. Upon heating, the material birefringence–governed brightness progressively overtook the structurally governed contribution, which was confirmed by voxel-wise brightness increasing monotonically with temperature (Fig. 5A). FFT analysis also showed broadening and splitting of spectral features upon heating, consistent with departure from the initial uniaxial arrangement due to voxel buckling (Fig. 5B). It is also demonstrated that the switching process between these two channels were reversible. The pixels exhibited a similar brightness trend over three heating-cooling (Fig. 5C). Using mapping functions *f*_1_(*x*) and *f*_2_(*x*), we then demonstrated switching between more complex images. At 25°C, the array displayed the Van Gogh’s self-portrait. Upon heating to 60°C, it transitioned gradually to the *Starry Night* (Fig. 5F and movie S2). Brightness analysis and magnified views confirmed that heating could induce voxel deformation and thus mesogen director reorientation, thereby activating the power-dependent contrast encoded in the material birefringence channel (Fig. 5, C and D).

## DISCUSSION

These examples illustrate seamless transitions between high-resolution images and highlight complex information encoding. This transition is governed by shrinkage-induced compressive forces, and the actuating stimulus is not restricted to temperature but can also arise from ionic, photonic, or other cues that induce hydrogel contraction (*47*–*49*). Blurred images may occur during switching, which arises from the continuous hydrogel phase transition and gradual voxel deformation. This intermediate state is transient rather than thermodynamically stable, and its duration can be reduced by faster thermal ramping or by narrowing the transition window through material optimization. The voxel density can surpass that of conventional electronic displays owing to the submicrometer tunability of voxel dimensions. Although 2PP is comparatively slow (∼13,000 voxels per hour for 10 μm by 10 μm), it provides precise control for elucidating the switching mechanism and establishing design rules. The present platform is preencoded and designed to demonstrate mechanically selectable dual-birefringence channels within a stable polymeric architecture. In principle, integrating dynamically tunable liquid-crystalline materials (e.g., light- or electroresponsive systems) could extend this framework toward reconfigurable or multistate optical operation beyond fixed dual encoding. As encoding remains purely geometric and dose based, selection remains purely mechanical, and the optical readout remains passive, this mechanism can, in principle, be implemented using scalable fabrication such as ultraviolet (UV) lithography or roll-to-roll imprinting. This strategy points toward sustainable display technologies powered by low-grade ambient stimuli such as temperature and humidity.

## MATERIALS AND METHODS

### Materials

*RM82*, NVP, and acrylamide (AAm) were bought from Energy Chemical. RM257 and 1-methyl-2-pyrrolidinone (NMP) were bought from Bide Pharmatech Ltd. and Sigma-Aldrich, respectively. Diphenyl(2,4,6-trimethylbenzoyl)phosphine oxide (TPO-L) and propylene glycol methyl ether acetate (PGMEA) were purchased from Aladdin. 2-Hydroxyethyl methacrylate (HEMA), *N*-isopropylacrylamide (Nipam), PEGDA (Mn550), polyethylene glycol (PEG, Mn400), and lithium phenyl-2,4,6-trimethylbenzoylphosphinate (LiPt) were obtained from 3A Senrise. Polymide (PI) and polystyrene (PS) microspheres were purchased from Sheng Man Wei and Rigor Science, respectively.

### 2PP for LCN voxels

Before 2PP, cells for aligning photoresists were first prepared. To prepare the alignment layer, glass slides were covered with a thin layer of PI via spin-coater at 800 rpm for 10 s to spread the solution uniformly, followed by 9500 rpm for 30 s to obtain a uniform thin layer. The coated slides were then baked at 180°C to solidify the PI layer. After that, the PI layer on the surface of the glass slide was unidirectionally rubbed against a velvet cloth to create aligned microgrooves, serving as the template for uniform liquid crystal orientation. The surface-imposed anchoring defines the nematic director throughout the film thickness and establishes a strong and uniform initial orientation. Two glass slides were assembled to obtain a cell with their PI-coated surfaces facing each other, using PS microspheres (diameter = 20 μm) as spacers and UV glue as bonding agent. For preparing the LCN, RM82 (74 mg), RM257 (50 mg), TPO-L (1 mg), and NVP (1 mg) were mixed and subsequently heated using a heat gun until fully liquefied. For 2PP, the liquefied LC precursor mixture was transferred into the prepared cell and subsequently underwent precisely printing process using a Nanoscribe system (Nanoscribe GmbH, Germany). Notably, the alignment direction of LC was mounted orthogonal to the 0° writing angle. 2PP is then performed within the prealigned LCN, and rapid cross-linking effectively locks in the director configuration. Even if the laser would have influence on the orientation of the LC (*34*), our LC is intrinsically anisotropic before curing due to nematic ordering. After printing, the upper glass slide was peeled off and the bottom slide with LCN was soaked in PGMEA for 10 min for developing the uncured LC. Considering that the brightness of LCN reaches the maximum at 45° CPC, the observation angle under nonzero CPC was set as 45° CPC in this work (fig. S1).

### Construction of complex patterns

To realize complex image encoding using 2PP, we developed a programmable fabrication logic, which allows independent parameter modulation at pixel level. DeScribe 2.7 was used to programme and generate codes for 2PP.

#### Image pixelation, segmentation, and block-wise printing sequence

Images were first pixelated into arrays of discrete square pixels (e.g., 200 by 200), and 10 by 10 pixels served as one printing block. A 10 μm–by–10 μm square was used as a basic printing voxel. Before printing each block, an interface searching command (FindInterfaceAt 0) was carried out to ensure accurate focal positioning. Within each block, the laser followed a serpentine scanning path defined by successive XOffset and YOffset movements to traverse all 100 pixels systematically. Upon completion of a block, the printing stage was shifted using MovestageX and MovestageY commands to initiate the next block. This process was repeated until the entire image array was fully printed.

#### Rainbow construction

Ten-micrometer cubes with hatching distance of 0.2 μm and writing angle of 0° serve as the printing units for construction of the colorful rainbow image. After pixelation, pixels of rainbow image in different colors were mapped to cubes with different height. To be specific, red, orange, yellow, green, cyan, blue, and white were mapped to cubes with height of 2.25, 2.00, 1.50, 4.00, 3.75, 3.25, and 1.00 μm, respectively.

#### Single-channel complex pattern construction

For brightness-based complex pattern construction, 10-μm cubes with hatching distance of 3.3 μm were used as basic printing unit and images first received grayscale before pixelation. For angle-dependent programming, pixel brightness values were normalized to [0,1] and mapped to corresponding writing angles in the range 0° to 45°, using a linear function *f*_1_(*x*), described as

$$f_{1}\left(x\right)=45x/255$$ (2)

Each angle-controlled voxel was printed by calling include cube_jobs\cube_{angle}_0_data.gwl, and the SP was set as 40 mW. These angle-varying cubes exhibited structure birefringence, leading to angle-dependent contrast under 0° CPC. For power mapping, brightness of pixels was mapped to SP in a range of power (e.g., 25 to 50 mW) via a function *f*_2_(*x*), described as

$$f_{2}\left(x\right)=50+50x$$ (3)

The printing command is Set $solidLaserPower = {power}, and the writing angle was set as 0°. This controlled printing dosage and thus the pixel height, modulating material birefringence under nonzero CPC.

#### Dual channel complex pattern construction

Brightness of pixels in image A (e.g., van Gogh’s self-portrait) was mapped to writing angle via *f*_1_(*x*). On top of the angle-encoded printing codes, image B (e.g., Starry Night) was encoded by mapping its normalized brightness to SP via a function *f*_2_(*x*). The printing command is Set $solidLaserPower = {power} and include cube_jobs\cube_{angle}_0_data.gwl*.*

### Hydrogel actuation of printed structures

The precursor mixture of thermo-responsive hydrogel were prepared according to previous work, which is composed of Nipam (500 mg), AAm (30 mg), HEMA (30 mg), PEGDA (200 mg), PEG (300 mg), LiPt (5 mg), and DI water (4.5 ml) (*46*). For embedding the printed structures, the bottom slide with printed LCN was used to make a secondary cell. Tape with thickness of 200 μm was used as spacers to separate the bottom glass slide with a new glass slide. Then, the precursor mixture of hydrogel was injected into the cell to fully cover the printed structures, followed by UV exposure for 15 min for polymerization. After peeling off the upper slide, the bottom slide was immersed in NMP for 10 min to dissolve the PI layer thus releasing the hydrogel with printed structure embedded. Next, the hydrogel was transferred into DI water to replace the NMP in the hydrogel network. For observing the behavior of the platform during temperature variations, the hydrogel was stored at a transparent and sealed chamber with a height of 300 μm and settled on precision heating stage.

### Characterization

A scanning electron microscope (Zeiss, Sigma 300) was used to observe the architecture of printed structures. An optical microscope (Olymbus, BX53) was used to observe the optical behavior of the printed structures. A spectrometer (Nikon Eclipse LV100 ND) was used to record the wavelength of printed structure. BRUKER Dimension Icon Scanning Probe Microscope was used to measure the thickness of printed structures.

## References

[1] J. K. Rowling, *Harry Potter Series* (Bloomsbury Publishing, 1997–2007).

[2] A. Santosa, Y. Chang, A. Dahlin, L. Österlund, G. Volpe, K. Xiong, Video-rate tunable colour electronic paper with human resolution. Nature 646, 1089–1095 (2025). 10.1038/s41586-025-09642-3 PMC12571869 41125884

[3] Q. Fu, W. Yu, G. Bao, J. Ge, Electrically responsive photonic crystals with bistable states for low-power electrophoretic color displays. Nat. Commun. 13, 7007 (2022). 10.1038/s41467-022-34745-0 PMC9669026 36385227

[4] C. Wang, R. Wu, H. Ling, Z. Zhao, W. Han, X. Shi, G. F. Payne, X. Wang, Toward scalable fabrication of electrochemical paper sensor without surface functionalization. NPJ Flexible Electron. 6, 12 (2022).

[5] Y. Ma, P. She, K. Y. Zhang, H. Yang, Y. Qin, Z. Xu, S. Liu, Q. Zhao, W. Huang, Dynamic metal-ligand coordination for multicolour and water-jet rewritable paper. Nat. Commun. 9, 3 (2018). 10.1038/s41467-017-02452-w PMC5760713 29317626

[6] S. Altay, E. Hoes, M. Wojcieszak, Following news on social media boosts knowledge, belief accuracy and trust. Nat. Hum. Behav. 9, 1833–1842 (2025). 10.1038/s41562-025-02205-6 PMC12454158 40579484

[7] F. Meng, J. Xie, J. Sun, C. Xu, Y. Zeng, X. Wang, T. Jia, S. Huang, Y. Deng, Y. Hu, Spreading dynamics of information on online social networks. Proc. Natl. Acad. Sci. U.S.A. 122, e2410227122 (2025). 10.1073/pnas.2410227122 PMC11789049 39847317

[8] R. Istrate, V. Tulus, R. N. Grass, L. Vanbever, W. J. Stark, G. Guillén-Gosálbez, The environmental sustainability of digital content consumption. Nat. Commun. 15, 3724 (2024). 10.1038/s41467-024-47621-w PMC11066053 38697974

[9] S. H. Kang, J. Y. Lee, J.-Y. Park, S.-G. Choi, S.-H. Oh, Y.-C. Joo, S.-K. Kang, Fully biodegradable electrochromic display for disposable patch. NPJ Flexible Electron. 8, 72 (2024).

[10] J. Min, Y. Jung, J. Ahn, J. Lee, J. Lee, S. H. Ko, Recent advances in biodegradable green electronic materials and sensor applications. Adv. Mater. 35, e2211273 (2023). 10.1002/adma.202211273 36934454

[11] C. Kant, A. Shukla, S. McGregor, S. Namdas, M. Katiyar, Large area inkjet-printed OLED fabrication with solution-processed TADF ink. Nat. Commun. 14, 7220 (2023). 10.1038/s41467-023-43014-7 PMC10632475 37940640

[12] Y. Wang, H. Nie, J. Han, Y. An, Y.-M. Zhang, S. X.-A. Zhang, Green revolution in electronic displays expected to ease energy and health crises. Light Sci. Appl. 10, 33 (2021). 10.1038/s41377-020-00455-9 PMC7867656 33550329

[13] C. Dai, Z. Li, Z. Li, Y. Shi, S. Zhang, Z. Li, Direct-printing hydrogel-based platform for humidity-driven dynamic full-color printing and holography. Adv. Funct. Mater. 33, 2212053 (2023).

[14] J. Yoon, C. Jung, J. Kim, J. Rho, H. Lee, Chemically and geometrically programmable photoreactive polymers for transformational humidity-sensitive full-color devices. Nat. Commun. 15, 6470 (2024). 10.1038/s41467-024-50876-y PMC11292010 39085253

[15] M. Rather, S. Vallabhuneni, A. J. Pyrch, M. Barrubeeah, S. Pillai, A. Taassob, F. N. Castellano, A. K. Kota, Color morphing surfaces with effective chemical shielding. Nat. Commun. 15, 3735 (2024). 10.1038/s41467-024-48154-y PMC11068873 38702308

[16] L. Cai, Y. Wang, L. Sun, J. Guo, Y. Zhao, Bio-inspired multi-responsive structural color hydrogel with constant volume and wide viewing angles. Adv. Opt. Mater. 9, 2100831 (2021).

[17] Y. Guo, J. Zhang, W. Hu, M. T. A. Khan, M. Sitti, Shape-programmable liquid crystal elastomer structures with arbitrary three-dimensional director fields and geometries. Nat. Commun. 12, 5936 (2021). 10.1038/s41467-021-26136-8 PMC8511085 34642352

[18] X. Zhang, T. Yin, J. Ge, Thermochromic photonic crystal paper with integrated multilayer structure and fast thermal response: A waterproof and mechanically stable material for structural-colored thermal printing. Adv. Mater. 36, e2309344 (2024). 10.1002/adma.202309344 37906731

[19] Y. Zheng, J. Chen, W. Mo, W. Hong, Thermo-responsive tri-state photonic crystals. Adv. Sci. 12, e06507 (2025). 10.1002/advs.202506507 PMC12407271 40464450

[20] K. M. Song, S. Kim, S. Kang, T. W. Nam, G. Y. Kim, H. Lim, E. N. Cho, K. H. Kim, S. H. Kwon, M. S. Jang, Y. S. Jung, Microcellular sensing media with ternary transparency states for fast and intuitive identification of unknown liquids. Sci. Adv. 7, eabg8013 (2021). 10.1126/sciadv.abg8013 PMC8443176 34524852

[21] S. Brandt, I. Pavlichenko, H. Patel, V. G. Portilla, M. Kolle, B. V. Lotsch, J. Aizenberg, M. Staymates, B. A. Craven, J. M. MacCrehan, J. A. Rogers, S. Sundaram, A. Vergara, R. Huerta, M. A. Burns, J. L. Moran, J. A. Smith, Nonequilibrium sensing of volatile compounds using active and passive analyte delivery. Proc. Natl. Acad. Sci. U.S.A. 120, e2303928120 (2023). 10.1073/pnas.2303928120 PMC10400973 37494398

[22] T. H. Ware, M. E. McConney, J. J. Wie, V. P. Tondiglia, T. J. White, Voxelated liquid crystal elastomers. Science 347, 982–984 (2015). 10.1126/science.1261019 25722408

[23] Y. Hu, C. Qi, D. Ma, D. Yang, S. Huang, Multicolor recordable and erasable photonic crystals based on on-off thermoswitchable mechanochromism toward inkless rewritable paper. Nat. Commun. 15, 5643 (2024). 10.1038/s41467-024-49860-3 PMC11226673 38969630

[24] Z. Zhou, X. Zhang, S. Halder, D. Yang, S. Huang, Smart switchable window with bistability and tunability of transmission. Adv. Opt. Mater. 12, 2302851 (2024).

[25] K. Xiong, O. Olsson, J. Svirelis, A. Dahlin, Video speed switching of plasmonic structural colors with high contrast and superior lifetime. Adv. Mater. 33, e2103217 (2021). 10.1002/adma.202103217 PMC11468514 34448507

[26] M. Wang, C. Nie, J. Liu, S. Wu, Organic–inorganic semi-interpenetrating networks with orthogonal light- and magnetic-responsiveness for smart photonic gels. Nat. Commun. 14, 1000 (2023). 10.1038/s41467-023-36706-7 PMC9946997 36813808

[27] F. Chun, B. Zhang, Y. Gao, X. Wei, Q. Zhang, W. Zheng, J. Zhou, Y. Guo, X. Zhang, Z. Xing, X. Yu, F. Wang, Multicolour stretchable perovskite electroluminescent devices for user-interactive displays. Nat. Photonics 18, 856–863 (2024).

[28] R. Huang, N. Cao, G. Gu, S. Wu, A bifunctional electrochromic/electrofluorochromic ionic liquid enables displayable smart windows with high energy efficiency. Adv. Funct. Mater. 35, e11224 (2025).

[29] G. Zhu, F. Sun, Y. Ji, H. Hu, M. Yang, Y. Zhang, X. Deng, Y. Zheng, C. Wei, D. Wang, Developing intelligent control of photoresponsive materials: From switch-type to multi-mode. Chem. Soc. Rev. 54, 7347–7376 (2025). 10.1039/d4cs00296b 40624955

[30] A. F. Demirörs, E. Poloni, M. Chiesa, F. L. Bargardi, M. R. Binelli, W. Woigk, L. D. C. de Castro, N. Kleger, F. B. Coulter, A. Sicher, H. Galinski, F. Scheffold, A. R. Studart, Three-dimensional printing of photonic colloidal glasses into objects with isotropic structural color. Nat. Commun. 13, 4397 (2022). 10.1038/s41467-022-32060-2 PMC9338281 35906208

[31] Y. Geng, R. Kizhakidathazhath, J. P. F. Lagerwall, Robust cholesteric liquid crystal elastomer fibres for mechanochromic textiles. Nat. Mater. 21, 1441–1447 (2022). 10.1038/s41563-022-01355-6 PMC9712110 36175519

[32] Y. Yao, M. Wilborn, B. Lemaire, F. Trigka, F. Stricker, A. H. Weible, S. Li, R. K. A. Bennett, T. C. Cheung, A. Grinthal, M. Zhernenkov, G. Freychet, P. Wąsik, B. Kozinsky, M. M. Lerch, X. Wang, J. Aizenberg, Programming liquid crystal elastomers for multistep ambidirectional deformability. Science 386, 1161–1168 (2024). 10.1126/science.adq6434 39636998

[33] M. Zhang, Y. Lee, Z. Zheng, M. T. A. Khan, X. Lyu, J. Byun, H. Giessen, M. Sitti, Micro- and nanofabrication of dynamic hydrogels with multichannel information. Nat. Commun. 14, 8208 (2023). 10.1038/s41467-023-43921-9 PMC10713606 38081820

[34] K. Hisano, M. Aizawa, M. Ishizu, Y. Kurata, W. Nakano, N. Akamatsu, C. J. Barrett, A. Shishido, Scanning wave photopolymerization enables dye-free alignment patterning of liquid crystals. Sci. Adv. 3, 1701610 (2017). 10.1126/sciadv.1701610 PMC5681215 29152567

[35] M. Liu, Q. Chen, W. Zhang, Z. Wu, X. Yan, Y. Liu, L. Ma, W. Hu, Y.-Q. Lu, D. Luo, Order parameter-programmed 2D-to-3D morphing in liquid crystal networks. Adv. Opt. Mater. 14, e02857 (2026).

[36] C. Chen, I. C. Khoo, Optical vector field rotation and switching with near-unity efficiency. Proc. Natl. Acad. Sci. U.S.A. 118, e2021304118 (2021). 10.1073/pnas.2021304118 PMC8072248 33853945

[37] G. Antonacci, R. Vanna, M. Ventura, M. L. Schiavone, C. Sobacchi, M. Behrouzitabar, D. Polli, C. Manzoni, G. Cerullo, Birefringence-induced phase delay enables Brillouin-background rejection. Nat. Commun. 15, 5202 (2024). 10.1038/s41467-024-49419-2 PMC11187154 38898004

[38] R. Gong, S. Tian, Y. Lei, Z. Zhang, Y. Xu, R. Lyu, F. Wang, H. Zhang, Z. Huang, C. Zhu, B. Liu, B. Ding, Tunable pure interference colors of 2D titania liquid crystal device. Sci. Adv. 11, eads0034 (2025). 10.1126/sciadv.ads0034 PMC11838015 39970223

[39] Y. Guo, H. Shahsavan, M. Sitti, Microscale polarization color pixels from liquid crystal elastomers. Adv. Opt. Mater. 8, 1902098 (2020).

[40] C. Koral, F. Bagci, A hybrid design for frequency-independent extreme birefringence combining metamaterials with the form birefringence concept. Photonics 11, 860 (2024).

[41] M. Chen, F. Fan, S. Xu, S. Chang, Artificial high birefringence in all-dielectric gradient grating for broadband terahertz waves. Sci. Rep. 6, 38562 (2016). 10.1038/srep38562 PMC5146933 27934962

[42] F. Li, S. Liu, W. Liu, Y. Zhang, X. Zhang, J. Liu, Z. Li, X. Wang, Y. Wang, H. Yang, Z. Chen, Q. Chen, Y. Song, X. Zhang, Y. Han, 3D printing of inorganic nanomaterials by photochemically assisted direct laser writing. Science 381, 1468–1474 (2023). 10.1126/science.adg6681 37769102

[43] H. Wang, W. Zhang, D. Ladika, H. Yu, D. Gailevičius, H. Wang, C.-F. Pan, P. N. S. Nair, Y. Ke, T. Mori, J. Y. E. Chan, Q. Ruan, M. Farsari, M. Malinauskas, S. Juodkazis, M. Gu, J. K. W. Yang, Two-photon polymerization lithography for optics and photonics. Adv. Funct. Mater. 33, 2214211 (2023).

[44] A. Wolfel, C. Johnbosco, A. Anspach, M. Meteling, J. Olijve, N. F. König, J. Leijten, Bioxolography using diphenyliodonium chloride and n-vinylpyrrolidone enables rapid high-resolution volumetric 3D printing of spatially encoded living matter. Adv. Mater. 37, e2501052 (2025). 10.1002/adma.202501052 PMC12447060 40285580

[45] P. Chansoria, M. Winkelbauer, S. Zhang, J. Janiak, H. Liu, D. Boev, A. Morandi, R. Grange, M. Zenobi-Wong, Structured light projection using image guide fibers for in situ photo-biofabrication. Adv. Mater. 37, e2419350 (2025). 10.1002/adma.202419350 PMC12243700 40297914

[46] M. Zhang, A. Pal, Z. Zheng, G. Gardi, E. Yildiz, M. Sitti, Hydrogel muscles powering reconfigurable micro-metastructures with wide-spectrum programmability. Nat. Mater. 22, 1243–1252 (2023). 10.1038/s41563-023-01649-3 PMC10533409 37604911

[47] C. Zheng, S. Jia, Bioinspired photoresponsive soft robotic lens. Sci. Robot. 10, adw8905 (2025). 10.1126/scirobotics.adw8905 PMC13049599 41124296

[48] H. Chen, F. Bian, Y. Wang, Y. Zhao, L. Shang, Colorimetric photonic tongue for metal ions screening. Matter 5, 1590–1602 (2022).

[49] X. Liu, M. Gao, J. Chen, S. Guo, W. Zhu, L. Bai, W. Zhai, H. Du, H. Wu, C. Yan, Recent advances in stimuli-responsive shape-morphing hydrogels. Adv. Funct. Mater. 32, 2203323 (2022).

