# Supplementary material for: Morphing polymer voxels for dynamic information switching
Source: Sci Adv. 2026 Sep 25;12(39):eaeg9993. doi: 10.1126/sciadv.aeg9993 (PMC13614403; doi:10.1126/sciadv.aeg9993)
Supplement: Supplementary file 1 [file sciadv.aeg9993_sm.pdf]

Science Advances
AAAS

Supplementary Materials for

Morphing polymer voxels for dynamic information switching

Lijun Cai *et al.*

Corresponding author: Joel K. W. Yang, joel_yang@sutd.edu.sg; Hong Liu, liu_hong@a-star.edu.sg; Mingchao Zhang, zhangmc@nus.edu.sg

Sci. Adv. **12**, eaeg9993 (2026)
DOI: 10.1126/sciadv.aeg9993

The PDF file includes:

Figs. S1 to S13
Legends for movies S1 and S2

Other Supplementary Material for this manuscript includes the following:

Movies S1 and S2

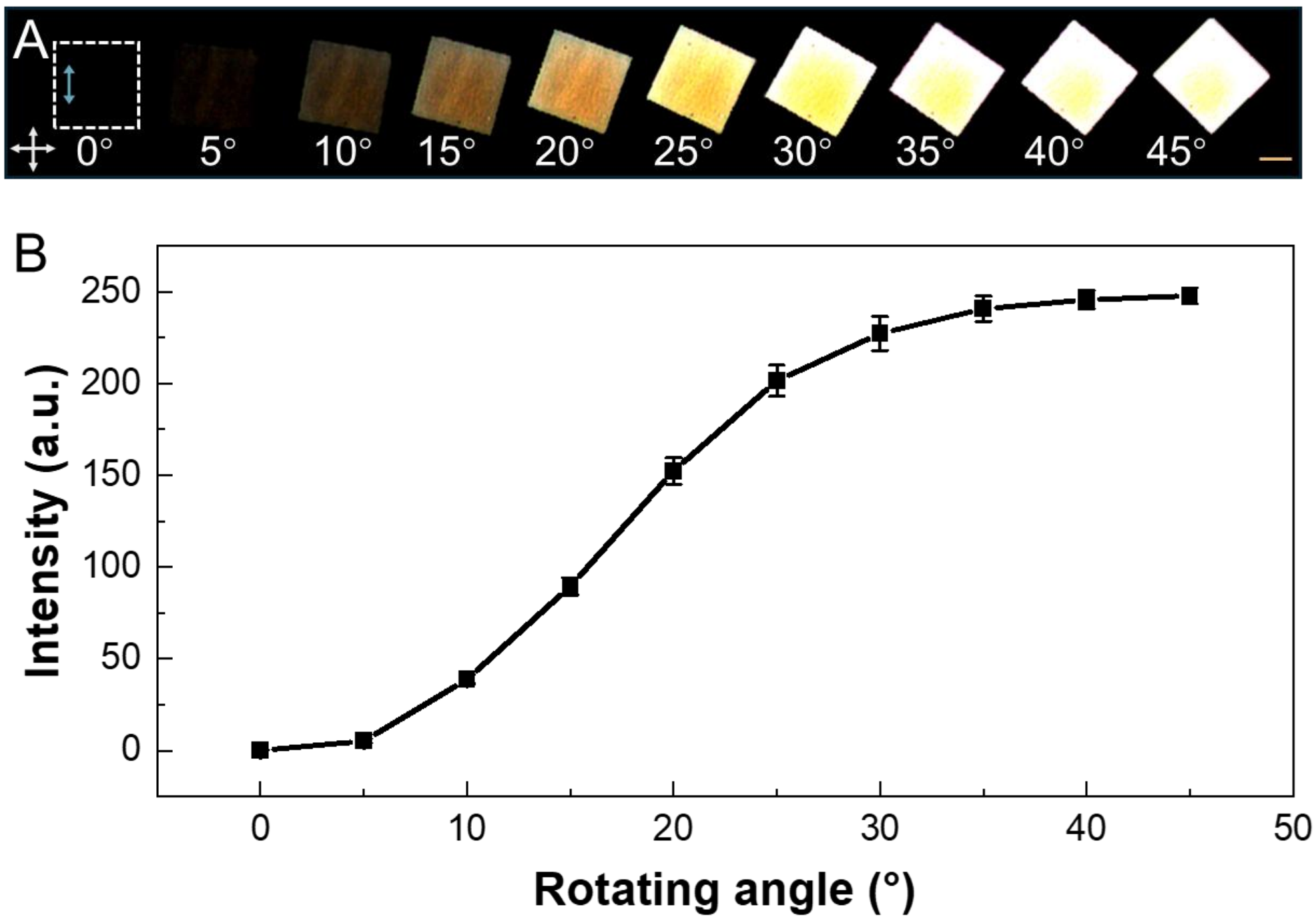

Fig. S1.

Polarized optical images of LCN voxels at different azimuth CPC (A) and their relative intensity (B), scale bar is 20 μm.

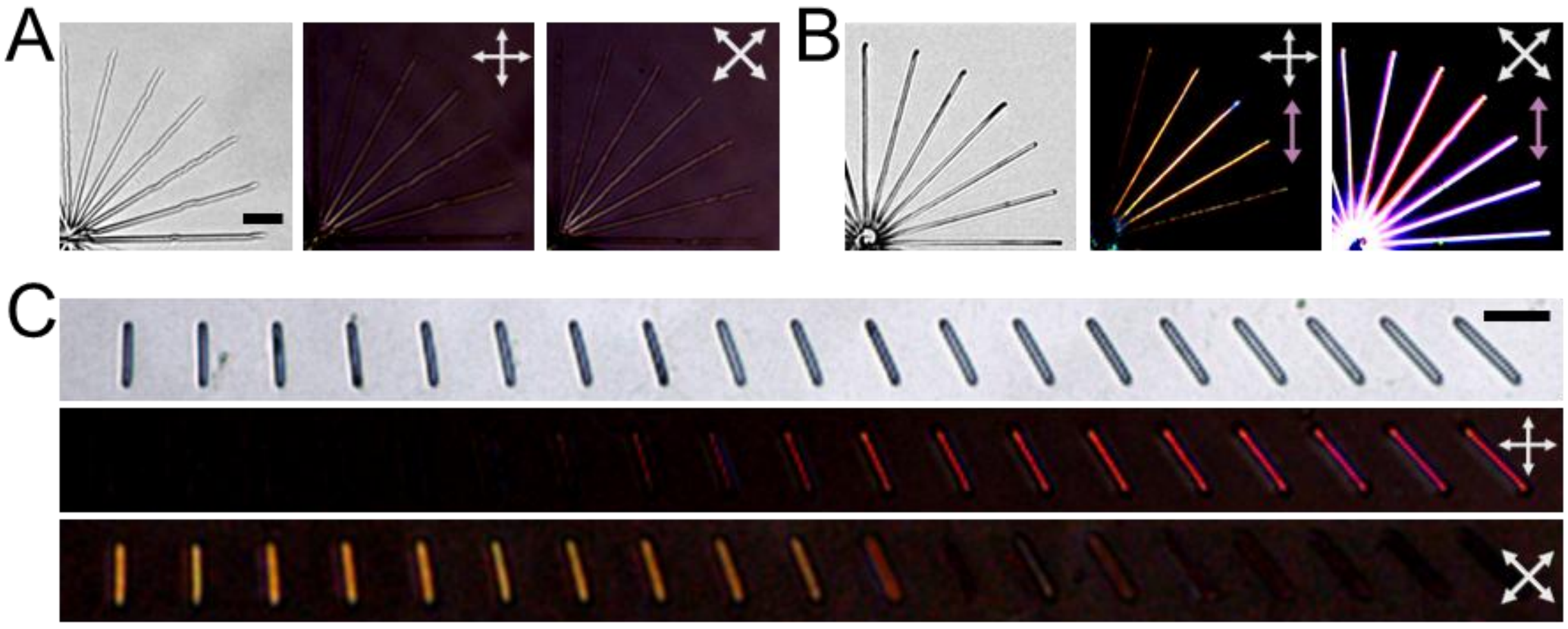

Fig. S2.

(A) Bright-field image and polarized images of PEGDA voxels. (B) Bright-field image and polarized images of LCN voxels. (C) Bright-field image and polarized images of PEGDA voxels with writing angles from 0° to 45°. Scale bars are 10 μm.

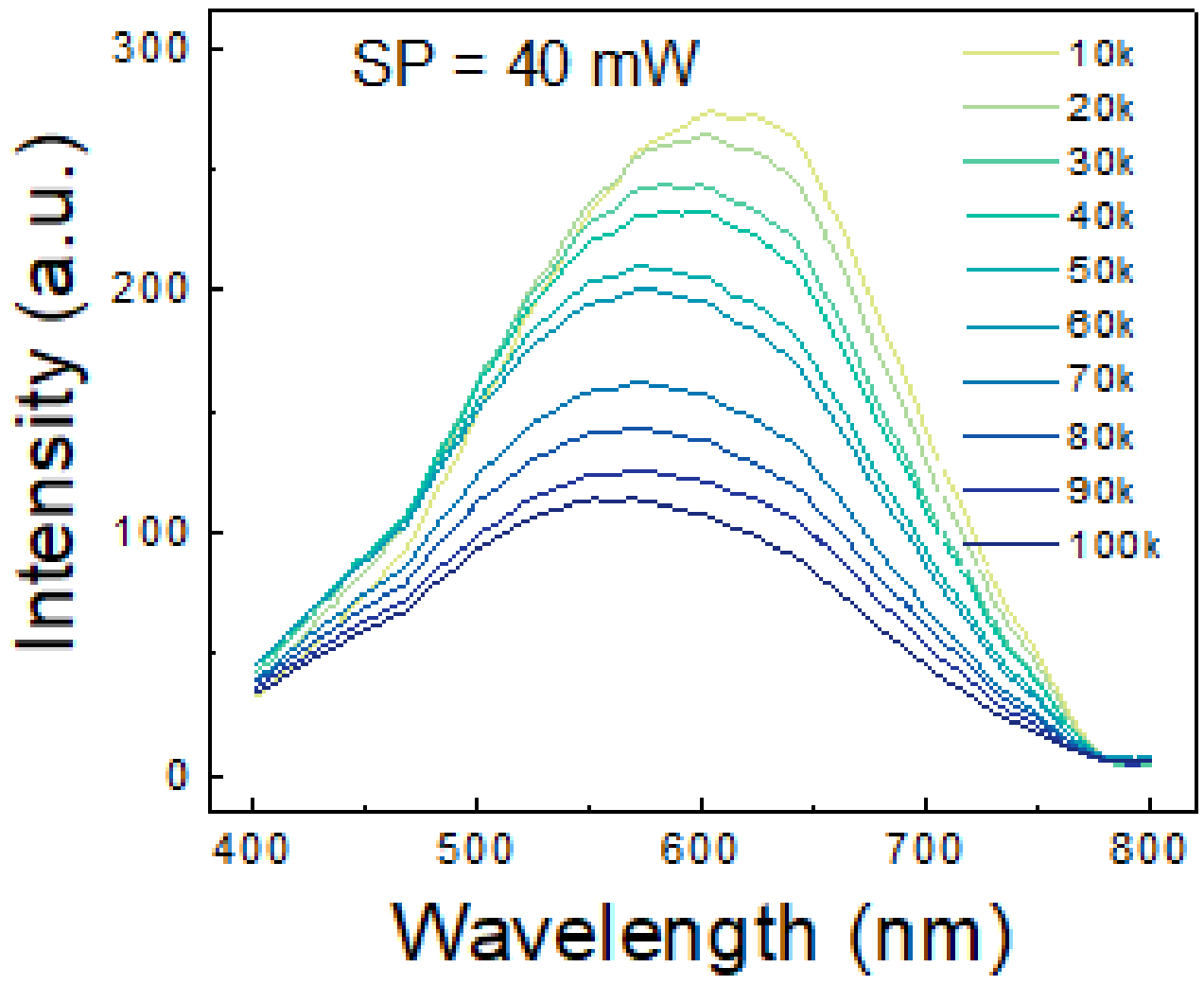

Fig. S3.
Spectrum of the LCN voxels printed with SP of 40 mW and different SS.

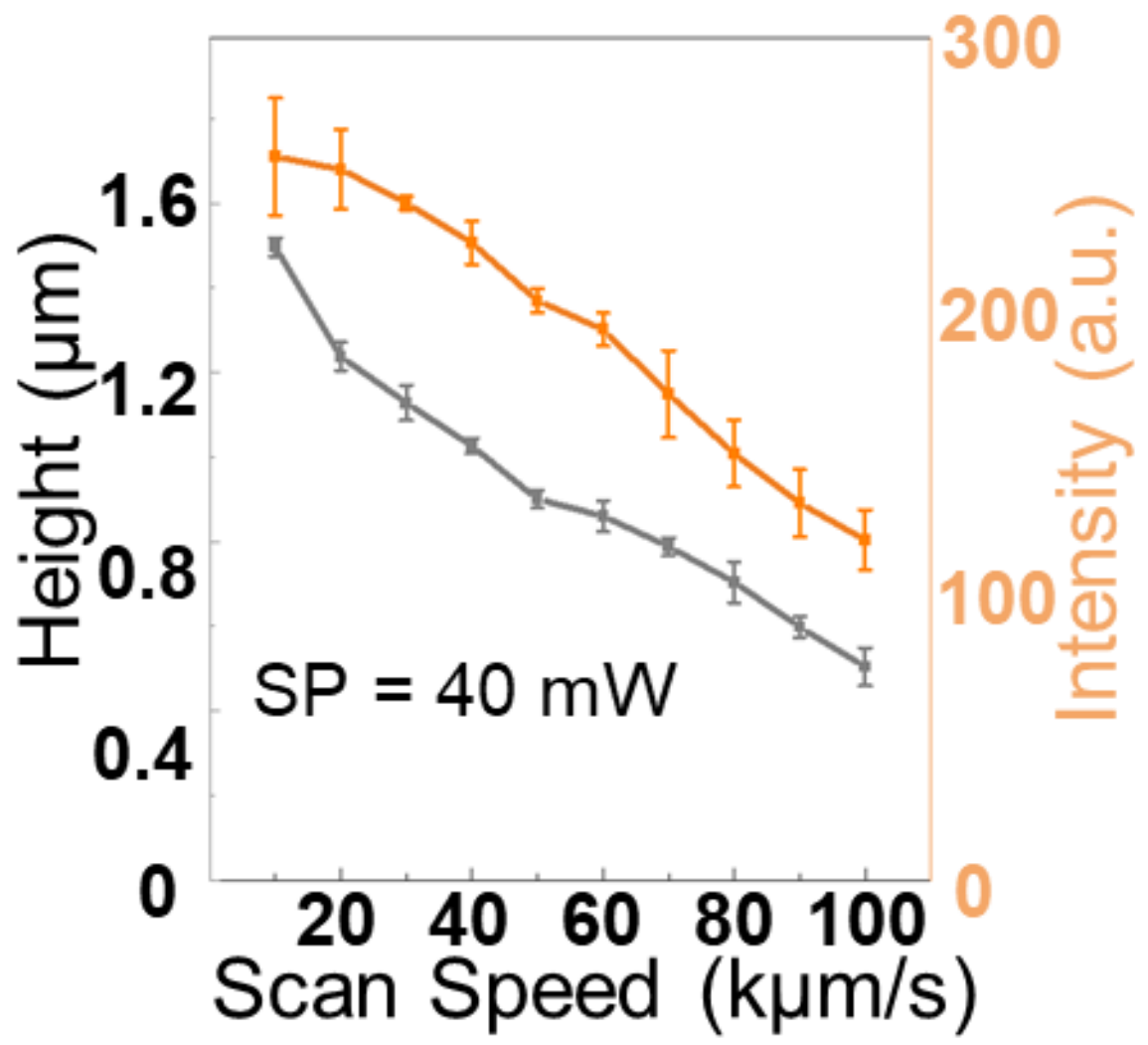

Fig. S4.
Height and intensity of LC cubes printed with laser power of 40 mW and different different scan speed.

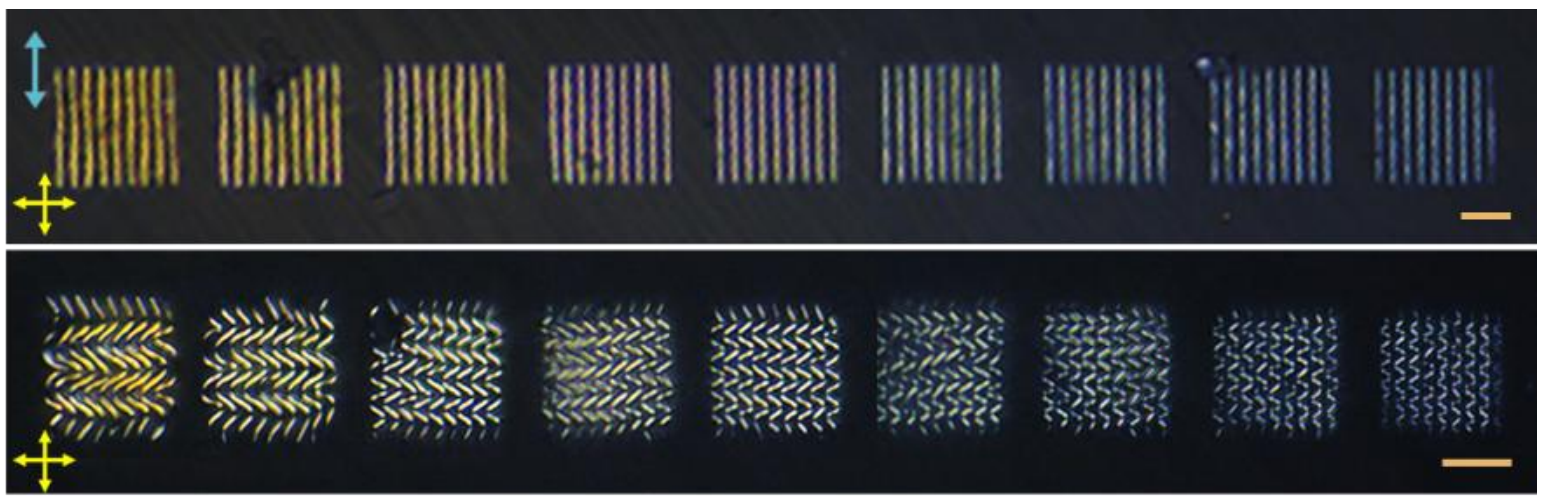

Fig. S5.
Polarized optical images of LC filamentary voxel printed with SS of 10 kμm/s and different SP before and after hydrogel-induced deformation. Scale bars are 20 μm.

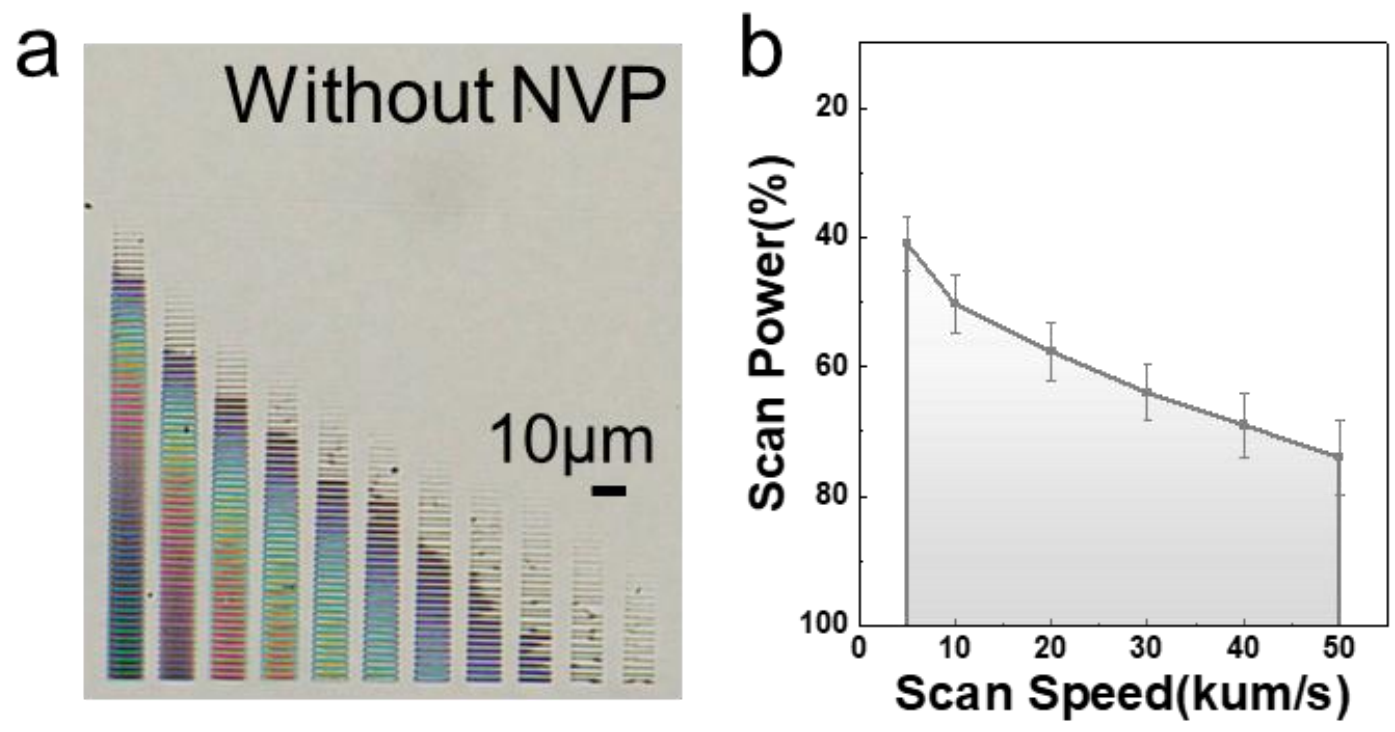

Fig. S6.
Printability of LC resist without NVP.

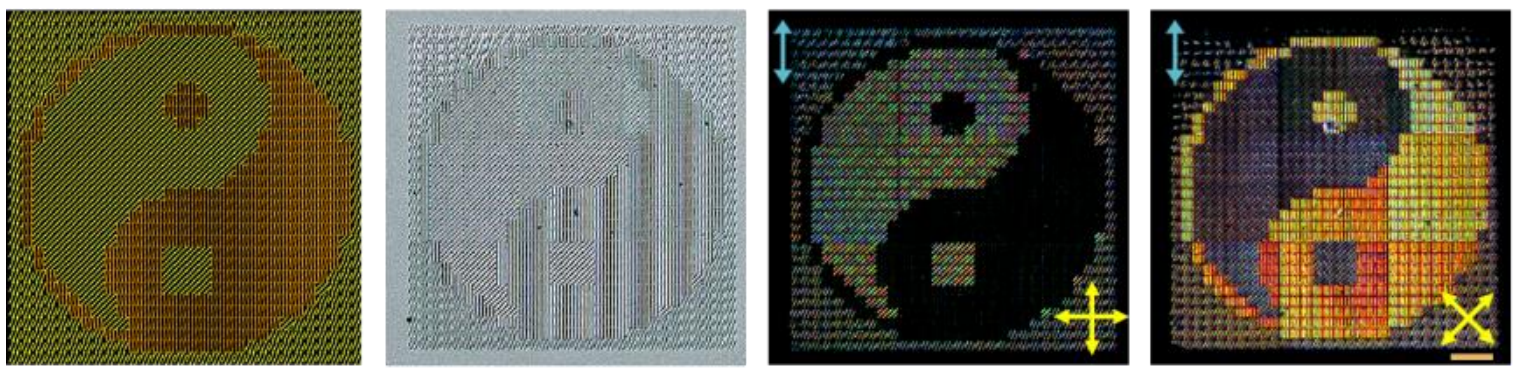

Fig. S7.
Dual channel programmed Tai Chi diagram whose brightness distribution can be converted by sample rotation. Scale bars are 40 μm.

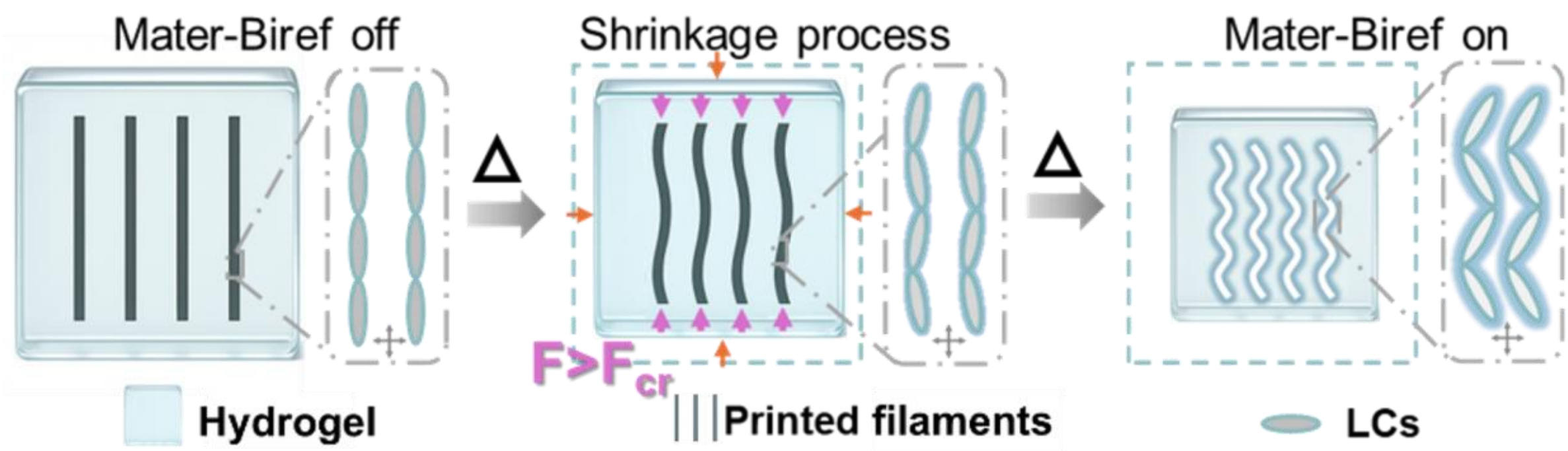

Fig. S8.
Schematic illustration of hydrogel-induced transformation and LC reorientation. Mater-Biref represents materials birefringence.

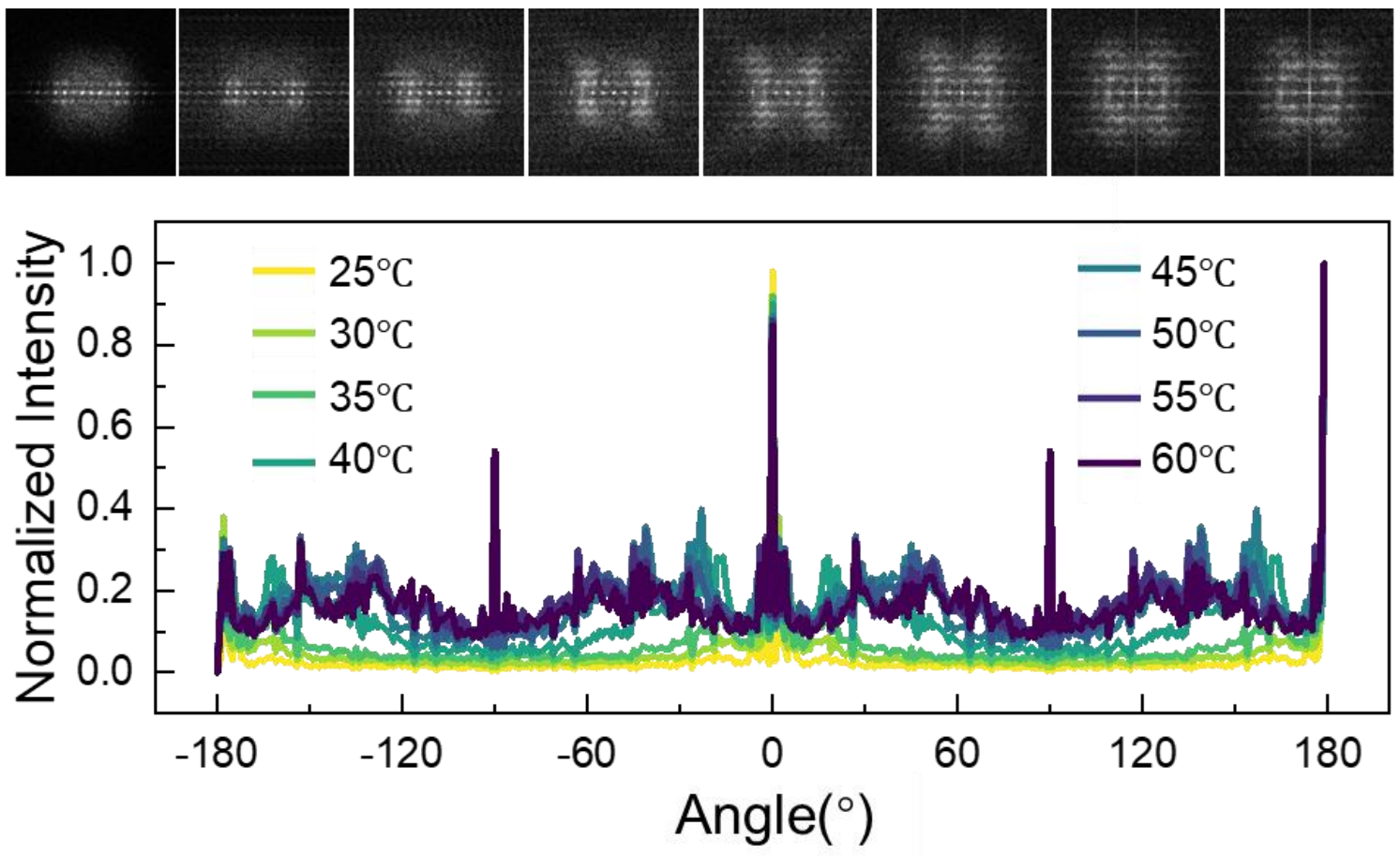

Fig. S9.
FFT magnitude spectrum and angular FFT of LC filamentary voxel upon heating process.

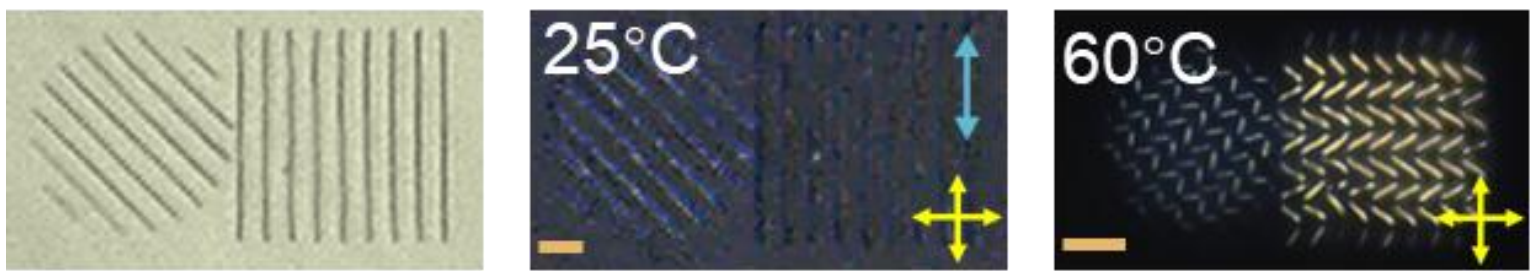

Fig. S10.
Bright field images, polarized images of printed LC round motif and square motif embedded in hydrogel at 25 °C and at 60 °C. Scale bars are 10 μm.

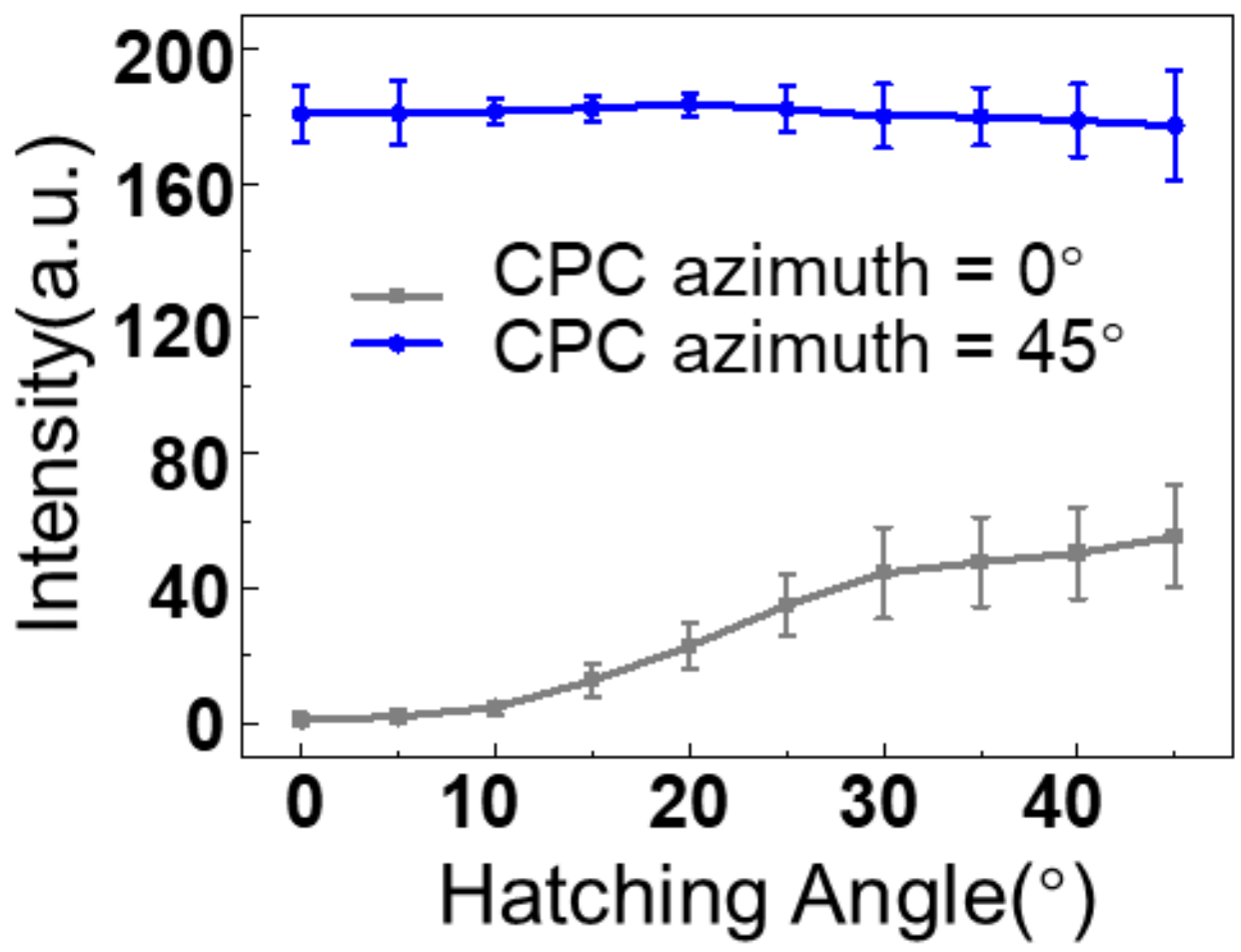

Fig. S11.
Brightness analysis of LC filamentary voxels at different azimuth CPC.

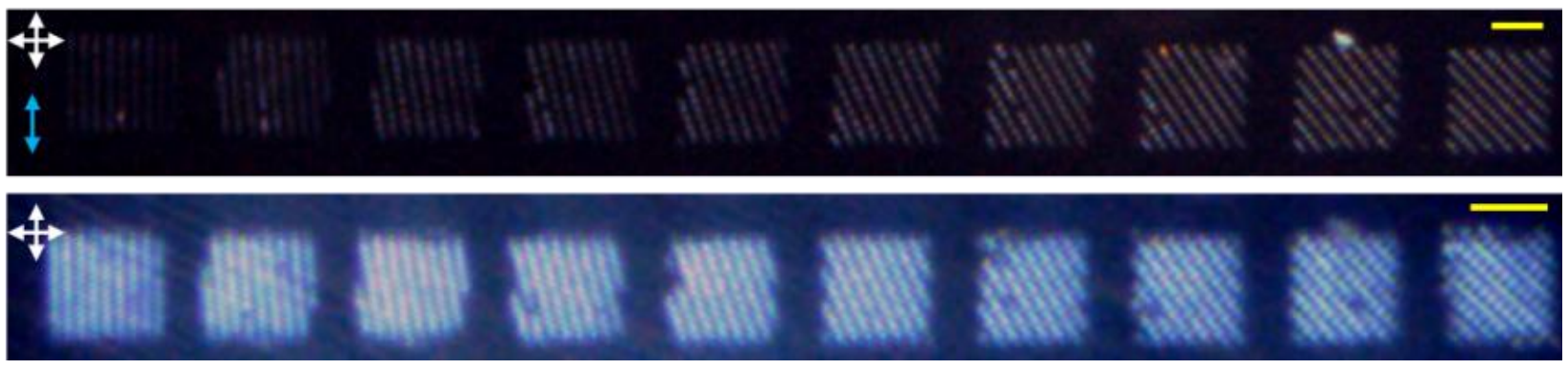

Fig. S12.
Representative images of fin arrays after the 10th heating–cooling cycles. Scale bars are 25μm.

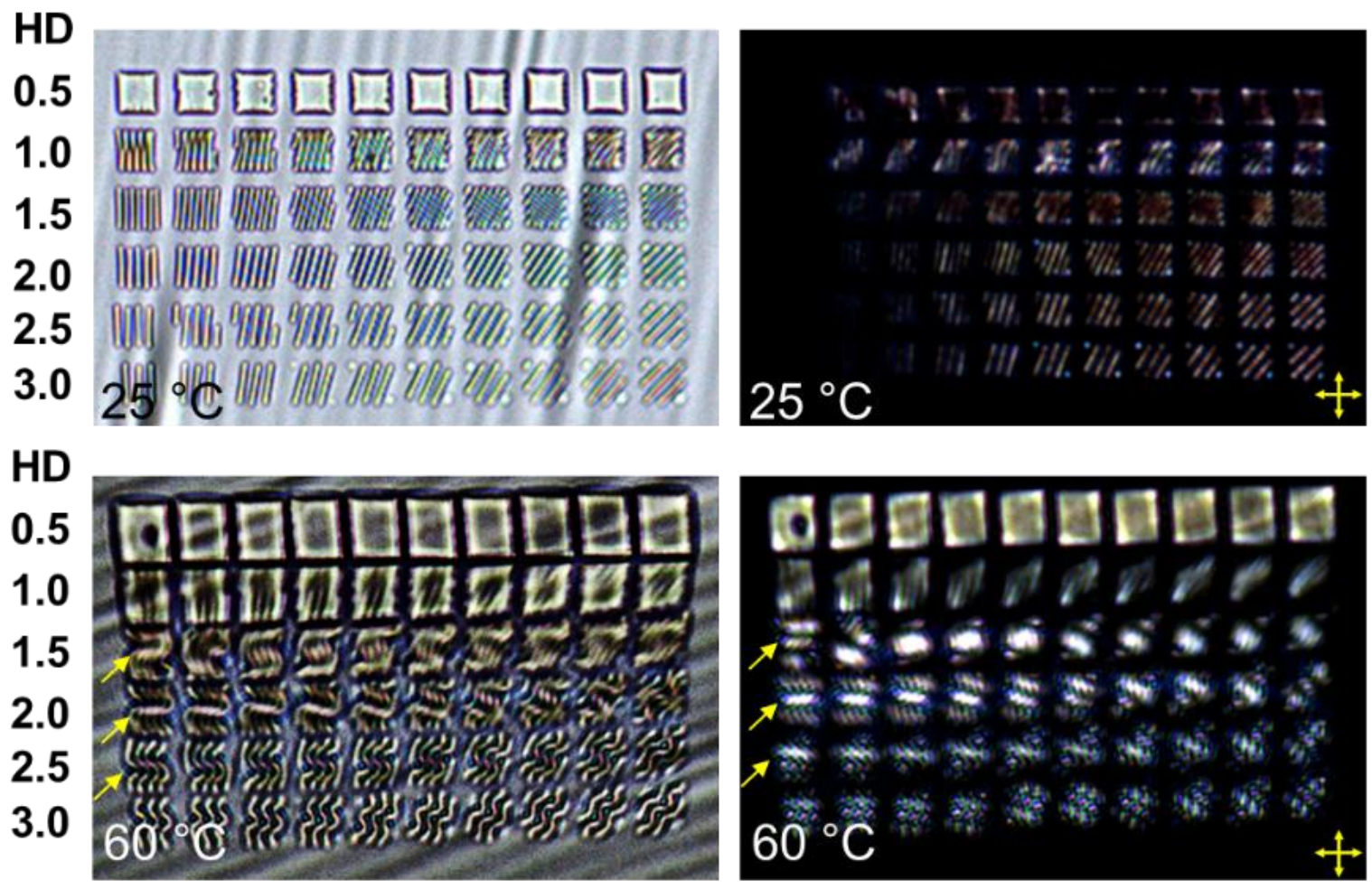

Fig. S13.
Optimization of hatching distance of 10 μm LC cubes. HD means hatching distance.

Movie S1.
Video showing the deformation of LC filamentary voxel arrays with different writing angles upon heating.

Movie S2.
Video showing the Vincent van Gogh portrait automatically, gradually and seamlessly switched to Starry night with temperature rising.
